# Platelet-Rich Plasma and Acellular Dermal Matrix in the Surgical Treatment of Hidradenitis Suppurativa: A Comparative Retrospective Study

**DOI:** 10.3390/jcm12062112

**Published:** 2023-03-08

**Authors:** Marcin Gierek, Agnieszka Klama-Baryła, Wojciech Łabuś, Beata Bergler-Czop, Kornelia Pietrauszka, Paweł Niemiec

**Affiliations:** 1 Dr Sakiel Center for Burns Treatment, Jana Pawła II Street 2, 41-100 Siemianowice Śląskie, Poland; 2 Department of Dermatology, Medical University of Silesia, Francuska Street, 40-027 Katowice, Poland; 3 Department of Biochemistry and Medical Genetics, School of Health Sciences in Katowice, Medical University of Silesia, Medykow Street 18, 40-752 Katowice, Poland

**Keywords:** platelet-rich plasma, hidradenitis suppurativa, acellular dermal matrix, split-thickness skin graft, surgical treatment

## Abstract

(1) Introduction: Hidradenitis suppurativa (HS) is an inflammatory skin disease with recurrent, chronic, painful, and purulent skin lesions. Topical or systemic antibiotics are the most widely used treatments for the management of mild stages of the disease. In chronic cases (Hurley II/III), wide excision of lesions should be considered. During reconstructive surgery, the most problematic aspect is wound closure. Very large excisional wounds require reconstructive techniques such as skin flaps, skin grafts, or both. Surgical methods have their limitations, so reconstructive methods in HS surgery need to be continuously improved through the use of, for example, platelet-rich plasma and acellular dermal matrix; (2) Methods: The aim of this study was to evaluate the clinical outcomes and efficacy of surgical treatment of patients with HS using local skin flaps injected with PRP compared to a group of local skin flaps without platelet-rich plasma injection, an acellular dermal matrix, and split-thickness skin graft co-grafts. Sixty-one patients (29 males and 32 females) were included in the study. Most patients were characterized by Hurley grade III HS; (3) Results: The use of PRP injection in reconstructions (skin flaps) improved healing and reduced the number of complications, a notable trend in this study. A co-graft of acellular dermal matrix and split-thickness skin graft gave better therapeutic results than split-thickness skin graft alone (fewer days in hospital, fewer postoperative complications); (4) Conclusions: PRP injected into skin flaps, co-grafted acellular dermal matrix, and split-thickness skin grafts are good options for the surgical treatment of hidradenitis suppurativa.

## 1. Introduction

Hidradenitis suppurativa (HS) is an inflammatory skin disease with a clinical presentation of characteristic recurrent, chronic, painful, and suppurating skin lesions [1,2]. HS is also known as acne inversa, and the first mentions of this disease date back to 1839 (Velpaeu) [3]. The worldwide incidence of HS has been documented in the range of 0.00033–4.1% [4,5]. Hidradenitis suppurativa has a significant impact on health-related quality of life. Anxiety disorders and depression are common in patients with hidradenitis suppurativa [6]. This devastating disease has a significant impact on many aspects of patients’ lives [7]. The cause of hair follicle plugging is still unclear and subject to scientific debate, although immune and genetic factors, particularly variants in genes encoding ɣ secretase complex proteins (*NCSTN* encoding nicastrin; *PSEN1* for presenilin-1; *PSENEN* for presenilin enhancer; gamma-secretase subunit), hormonal fluctuations, and environmental risk factors are believed to play a role [8,9,10]. The most common sites for hidradenitis suppurativa are the main folds of the body, including the axillary and inguinal regions, the skin of the buttocks, the sub-mammary area, and the neck, waist, and inner side of the thighs [11]. Hidradenitis suppurativa often has a very late diagnosis. An average delay of 7–10 years between the onset of the disease and its diagnosis has been reported [6]. HS has a mild, moderate, and severe course. The severity of the disease is assessed according to several validated scores, among which are the Hurley scale and the IHS4 (International Hidradenitis Suppurativa Severity Score) scale [1,2,3,4,5,6,7].

Ultrasound is a good method of assessing the severity of the disease. Ultrasound can increase the sensitivity of assessment compared to clinical examination [11]. For example, ultrasound can also be used to assess the effectiveness of treatment with biologic drugs [12].

Treatment with topical or systemic antibiotics is widely used as first-line therapy in the management of mild stages of the disease. In severe cases (Hurley II/III), surgical methods should be considered [1,13,14]. It has been proven that health-related quality of life significantly improves after surgical treatment [6]. The most important aspect of surgical treatment of HS is wide excision of HS lesions. During HS reconstructive surgery, the most problematic aspect is wound closure due to wide excision (very large excision wounds). Such cases require reconstructive techniques that are standard in such cases, such as skin flaps, skin grafts, or both [13,14]. Surgical reconstructive techniques for the treatment of HS are limited. Healing by secondary intention is widely recognized as an option for first-line surgical treatment, but there are also reconstructive surgery methods that can be used successfully in closing large wounds after wide excisions. There is no ideal method of wound closure in HS surgery. Local skin flaps (rotational skin flaps) appear to be a good method in terms of tissue coverage; however, they can cause displacement of potentially diseased tissue, which can lead to recurrence (hidradenitis suppurativa) [13,14]. Skin flaps are associated with the possibility of greater scarring and the possibility of vascular complications, such as necrosis of the skin flap. Split-thickness skin grafts can result in inflexible contracture scars, which can cause contractures, for example, in the armpits [14,15].

One of the more effective methods of treating HS is the surgical method, which has proven to have a positive effect on health-related quality of life after extensive surgical treatment [7]. Surgical methods have their limitations, so reconstructive methods in HS surgery need to be continuously improved.

Platelet-rich plasma (PRP), obtained from the patient’s blood, primarily contains concentrated platelets and other blood components, so it releases a high concentration of various bioactive molecules stored in granules, which can stimulate angiogenesis. Numerous papers prove the wide application of PRP in tissue regeneration in various fields, such as dentistry, orthopedics, maxillofacial surgery, sports medicine, ophthalmology, and peripheral nerve regeneration. Currently, the most extensive research is being conducted on the use of PRP for tissue regeneration and repair and the prevention of infection [16,17].

In reconstructive surgery, we often have to deal with large tissue defects. This creates the need to look for materials that are non-immunogenic and offer the possibility of filling tissue after excisions. Acellular dermal matrix (ADM) is a biological collagen matrix without immunogenicity that is more commonly used in surgical treatment. Reconstructive surgery is still looking for various biocompatible materials that could be widely used in surgery. Available biomaterials have their advantages and disadvantages [18]. The co-graft of acellular dermal matrix (ADM) and split-thickness skin graft (STSG) has shown good efficacy as an improved treatment for wide HS excision sites [19]. This study is a presentation of the treatment results of PRP-assisted reconstructive skin flap surgery methods and the results of ADM and STSG co-graft surgery.

The aim of the study was to evaluate the clinical outcomes and efficacy of surgical treatment of patients with hidradenitis suppurativa using local skin flaps injected with PRP compared to a group of local skin flaps without PRP injections and co-graft ADM and STSG.

## 2. Materials and Methods

In this retrospective comparative study, we assessed the impact of HS risk factors, comorbidities, disease severity, and surgical techniques on the efficacy of therapy in patients treated surgically for HS. A total of 61 patients surgically treated at the Dr. Sakiel Center for Burns Treatment in Siemianowice Slaskie, Poland between 2019 and 2022 were included in the study, including 24 patients with local skin flaps and the PRP-assisted method and seven patients with a co-graft of ADM and STSG. In addition, 26 patients were operated on with the classical method (skin flap reconstruction), and five patients with STSG alone were included in the study. The study was conducted in accordance with the Declaration of Helsinki and approved by the Institutional Ethics Committee of the Silesian Medical Chamber in Katowice, protocol code 30/2021 of 22 September 2021 (date of approval). The study was conducted according to the STROBE guidelines (Table S1).

**Figure 1:**
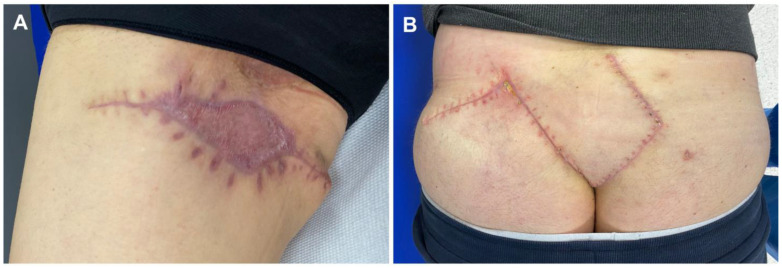
Completely healed patients after surgical treatment of hidradenitis suppurativa: (**A**) patient with a 6-month follow-up post-co-graft of ADM and STSG (right groin); (**B**) patient with a 6-month follow-up post-skin flap reconstruction with PRP injections. Legend: ADM, acellular dermal matrix; STSG, split-thickness skin graft; PRP, platelet-rich plasma.

Patients were treated with surgical methods available at our center (including a tissue bank). Patients were then followed up at the surgical outpatient clinic, with follow-up after 2 weeks and 6 months. Basic data were collected from the patients for analysis, such as age, sex, BMI, comorbidities, duration of the disease, time from first symptoms to proper diagnosis, location of lesions, and addictions. We included both male and female patients with hidradenitis suppurativa I-III grades on the Hurley Scale. Exclusion criteria: pregnant women, breastfeeding women, cancer in the last 5 years—presence of basal cell carcinoma (BCC), current biological and pharmacological treatment for HS, current anti-inflammatory or anti-platelet medications. Surgical treatment consisted of wide excision of lesions located in the armpits, groin, buttocks, and ano-genital regions (Figure 1).

### 2.1. Platelet-Rich Plasma (PRP)-Assisted Skin Flaps and the Process of PRP Preparation

**Figure 2:**
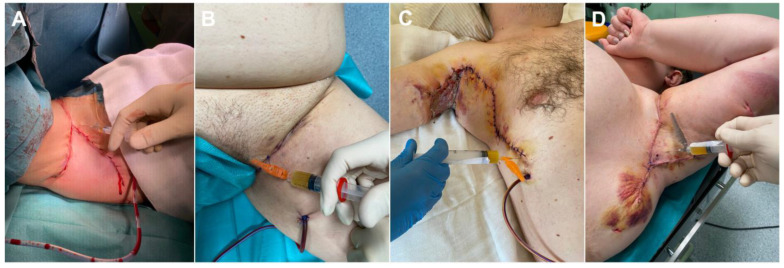
Platelet-rich plasma injections: (**A**) and (**B**) intraoperative images—injections after flap fixation; (**C**) PRP injection to local skin flap—1st day post-surgery (right axilla); (**D**) PRP injection to local skin flap—3rd day post-surgery (left axilla). Legend: PRP, platelet-rich plasma.

Under standardized conditions (in a treatment room equipped with disposable equipment, the same exposure to light), blood was collected, separated, and PRP was injected. PRP was injected into the local skin flap after reconstruction immediately after surgery and on postoperative days 1, 2, and 3. An Arthrex autologous conditioned plasma (ACP) double syringe (Arthrex GmbH, München, Germany) was used for plasma extraction. PRP was separated from fresh whole blood immediately after blood collection. The ACP syringes from Arthrex (Arthrex GmbH, München, Germany) were each filled with 1.5 mL of anticoagulant: sodium citrate (Citra-Lock^®^ 4% from Dirinco B.V. Ketelmeer 1, The Netherlands). For the preparation of PRP, 15 mL of venous blood was collected from the patients into an Arthrex ACP double syringe. The blood was centrifuged once for 5 min at 1500 rpm. Once the blood had been separated by centrifugation into a fraction for use in patients, the top layer of plasma above the red blood cell layer was collected. Immediately after centrifugation, fresh PRP was injected into the area of common extensor origin in a volume of 2.0–3.0 mL with a 1.2 mm needle. The first injection, “day 0,” was immediately after fixation of the local skin flap during the surgery. This treatment was continued on days 1, 2, and 3 postoperatively (during the dressing change). After the PRP injection, each patient was observed for 30 min for possible complications. Particular attention was paid to local inflammation and allergic reactions. On the day of PRP injection, a laser speckle contrast analysis examination was undertaken (Figure 2).

### 2.2. Co-Graft of Acellular Dermal Matrix and Split-Thickness Skin Graft

The ADM we used was our own in-house ADM, obtained from cadavers as allografts and then enzymatically processed in our Tissue Bank laboratory at the Burn Treatment Center in Siemianowice Slaskie. Human skin samples (intended for ADM preparation) were collected from multi-organ/multi-tissue deceased human donors (collection sites included such areas as the arms, forearms, thighs, shins, calves, chest, bottom, and back). A battery dermatome (Acculan III, Aesculap AG, Tuttlingen, Germany) was used. Skin depth was set to parameter 4–5 on the dermatome scale (0–14 scale). All storage, transport to the tissue bank, and preparation of allogeneic human skin grafts were carried out in accordance with the relevant standard operating procedures (SOP) of the tissue bank at the Dr. Sakiel Center for Burn Treatment in Siemianowice Śląskie, Poland. Briefly, tissue engineering (preparation) of allogeneic human Acellular Dermal Matrix (ADM) involves washing skin grafts in hypotonic solution, separating the dermis from the epidermis (1–3 h incubation in 2.4 µ/mL Dispase II, Gibco/Thermo Fisher Scientific, New York, NY, USA), and 24 h incubation of human dermis in 0.05% trypsin solution in EDTA (Life Technologies, Carlsbad, CA, USA); the enzyme solution was changed after 12 h incubation. After the decellularization process, uneven ADM fragments were cut off and the graft area was measured. Each graft was then registered in the ISBT 128 computer system and packed into triple bags. ADMs were sterilized with an electron beam of 35 kGy. The sterility of ADMs was confirmed by microbial culture. The donors were HIV, HCV, and Hepatitis B negative (which was confirmed before blood donation). ADMs obtained in this way were transplanted into wounds. After wide excision of the HS lesion, wounds were covered with ADM mesh in a ratio of 1:1.5. After ADM fixation, skin for grafting from the thighs at a ratio of 1:1.5 was harvested. STSG was transplanted directly onto the ADM layer.

### 2.3. Laser Speckle Contrast Analysis

**Figure 3:**
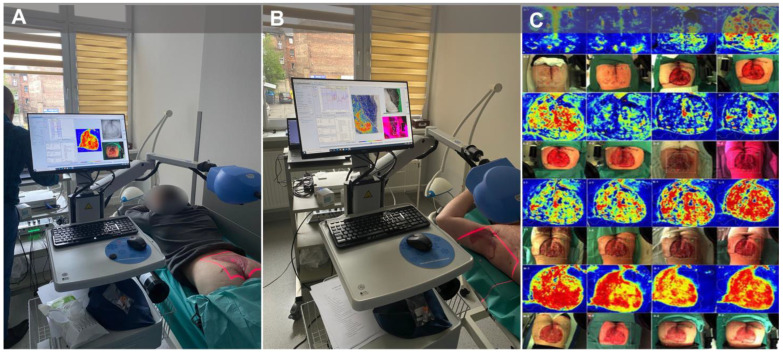
Laser speckle contrast analysis examinations. (**A**) patient during the LASCA examination after co-graft of ADM and STSG surgery (buttocks); (**B**) patient during the LASCA examination after co-graft of ADM and STSG surgery (right axilla); (**C**) microcirculatory perfusion and the healing process over a period of time (patient with co-graft of ADM and STSG to the buttocks). Legend: LASCA, laser speckle contrast analysis; ADM, acellular dermal matrix; STSG, split-thickness skin graft.

Laser speckle contrast analysis (LASCA), also known as laser speckle contrast imaging (LSCI), is a method that instantly visualizes microcirculatory tissue blood perfusion. It is an imaging technique that combines high resolution with high speed. When an object is illuminated by laser light, the backscattered light will form an interference pattern consisting of dark and bright areas. This pattern is called a speckle pattern. If the illuminated object is static, the speckle pattern is stationary. When there is movement in the object, such as red blood cells in a tissue, the speckle pattern will change over time. A camera with a fixed exposure time will record these changes in the speckle pattern as motion blur. The LASCA examination was performed on specific days: before admission, immediately before the procedure, immediately after the procedure, and in the days following the procedure. The measuring device was placed 30 cm from the test object. The examination lasted 20 s, with an image width of 20.1 cm and a height of 19.0 cm; 16 frames, 1 color photo per 10 s, and a resolution of 0.78 mm. LASCA examinations were undertaken before the patient’s admission to the hospital, during the operation, and immediately after, at days 1, 2, and 3 post-surgical treatment, 2 weeks post-surgical treatment, and 6 months post-surgical treatment. With LASCA, we were able to monitor the healing process, assess the effectiveness of surgical treatment, and detect vascular anomalies (such as skin flap ischemia) early (Figure 3).

### 2.4. Statistical Analysis

Statistica 13.0 software (TIBCO Software Inc., Carlsbad, CA, USA) was used for all statistical analyses carried out for the current work. The normality of the distribution was analyzed using the Shapiro-Wilk test, and all quantitative variables had a non-normal distribution. Spearman’s rank correlation coefficient (r_s_) was used as a measure of the correlation of quantitative variables. The Mann-Whitney U test or the Kruskal-Wallis test were used to compare quantitative data between grouping variables. All quantitative data were presented as the median and their spread as quartile deviation (QD). The *χ*^2^ test was used for analyses of qualitative data. For subgroups of fewer than ten subjects, the Yates’ correction was applied. Patients with missing data were rejected from the respect comparisons. Statistical significance was assumed for results at the level of *p* < 0.050. The *p* values of multiple comparisons were corrected using the Bonferroni correction. Study size and power analysis testing were not performed due to the rarity of the disease and the small group size.

## 3. Results

### 3.1. General and Clinical Characteristics of the Study Group

Baseline characteristics of the surgically treated patients were collected, including age, sex, body mass index, blood parameters, Hurley scale disease severity, lesion location, time since first symptoms, duration of disease, comorbidities, type of surgical intervention, and follow-up in the general surgery clinic 2 weeks after surgery and 6 months after surgery (Table 1).

### 3.2. Factors Affecting Clinical Phenotype and Complications

There was a statistically significant (*p* < 0.050) positive correlation between patients’ age and body mass index (BMI), disease duration, and time of diagnosis. The BMI value additionally correlated with disease duration and time of diagnosis, while disease duration correlated with time of diagnosis (Table 2).

Subjects with treatment complications during follow-up at week 2 after surgery had a higher weight than those without complications (103.50 ± 11.50 vs. 89.00 ± 12.50, *p* = 0.012), with differences in BMI showing no statistical significance (*p* = 0.117). Those with complications also had a lower white blood cell count (7.55 ± 1.34 vs. 9.80 ± 2.42, *p* = 0.024). Other parameters did not differentiate between subjects with and without complications at the 2-week follow-up (*p* > 0.050). At the 6th month of follow-up, complications were associated with a longer duration of hospitalization (8.00 ± 1.00 vs. 5.00 ± 1.50, *p* = 0.018). Other parameters did not differentiate between subjects with and without complications at the 6-month follow-up (*p* > 0.050).

Overweight/obese (BMI ≥ 25) patients were characterized by their older age (38.00 ± 7.00 vs. 31.00 ± 6.00, *p* = 0.020), higher BMI (33.00 ± 2.35 vs. 23.00 ± 1.00, *p* = 0.000), disease duration (10.00 ± 4.50 vs. 6.00 ± 2. 00, *p* = 0.010), longer time to its diagnosis (7.00 ± 4.50 vs. 5.00 ± 2.00, *p* = 0.014), higher hemoglobin levels (13.20 ± 1.15 vs. 12.40 ± 0.90, *p* = 0.010) and MCH (29.40 ± 2.00 vs. 27.70 ± 2.05, *p* = 0.022) relative to patients without overweight/obesity. The same parameters differentiated patients with obesity from those without, except for hemoglobin levels (*p* = 0.080). Patients with diabetes were characterized by older age (42.00 ± 4.00 vs. 35.00 ± 9.00, *p* = 0.024), higher BMI (33.00 ± 1.15 vs. 29.95 ± 4.63, *p* = 0.036), and higher MCH (30.60 ± 1.40 vs. 28.30 ± 1.88, *p* = 0.003) relative to patients without diabetes. Patients with metabolic disease had a higher BMI (33.70 ± 1.30 vs. 29.95 ± 4.58, *p* = 0.008), higher PLT levels (379.00 ± 22.50 vs. 316.95 ± 34.00, *p* = 0.034), and higher MCH (31.40 ± 1.03 vs. 28.55 ± 1.80, *p* = 0.002) relative to patients without metabolic disease. Hypertensive patients were characterized by older age (39.00 ± 6.00 vs. 32.50 ± 9.50, *p* = 0.007), higher BMI (33.30 ± 2.00 vs. 28.00 ± 5.00, *p* = 0.001), disease duration (13. 00 ± 4.00 vs. 6.50 ± 3.00, *p* = 0.011), higher hemoglobin levels (13.35 ± 1.10 vs. 12.40 ± 1.50, *p* = 0.043), and MCH (29.65 ± 1.45 vs. 27.95 ± 1.95, *p* = 0.013) relative to patients without hypertension. Patients who smoked cigarettes had a lower BMI (23.00 ± 5.56 vs. 31.20 ± 3.15, *p* = 0.017), higher PLT levels (396.00 ± 140.50 vs. 317.00 ± 29.50, *p* = 0.049), and longer hospitalization times (7.00 ± 2.00 vs. 5.00 ± 1.00, *p* = 0.008) relative to non-smokers. Patients with hypothyroidism had lower height (164.00 ± 2.50 vs. 176.00 ± 6.00, *p* = 0.003) and weight (87.00 ± 9.50 vs. 94.00 ± 13.50, *p* = 0.046), as well as higher platelet levels (343.00 ± 31.00 vs. 315.00 ± 39.00, *p* = 0.039) relative to patients without hypothyroidism.

The presence of an avascular anomaly on LASCA was not associated with patients’ age (*p* = 0.517), BMI (*p* = 0.878), duration of disease (*p* = 0.861), time of diagnosis (*p* = 0.478), or time of hospitalization (*p* = 0.878). Blood parameters also did not differentiate patients with the anomaly from those without it (*p* > 0.050).

### 3.3. Hurley Scale, Clinical Phenotype, and Complications

Factor analysis of the Hurley Scale revealed that patients with stage III disease were significantly older than patients with stage I disease and had significantly lower whole blood PLT levels than patients with stage I (Table 3).

The longest time of disease diagnosis, from the onset of first symptoms to diagnosis, as well as the longest duration of disease, characterized patients with stage II-HS, followed by those with stages III and I. Statistically significant differences in both parameters were observed between patients with stage I and stage III disease (Table 3).

**Figure 4:**
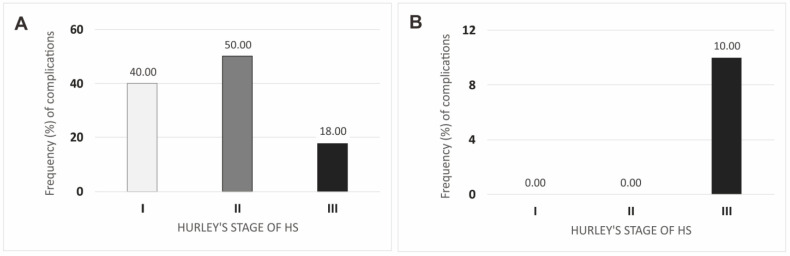
Complications after surgical interventions in regard to Hurley’s stage of Hidradenitis suppurativa in two-week (**A**) and six-month (**B**) follow-up. Legend: HS, hidradenitis suppurativa.

In the present study, we investigated whether the severity of the disease affected the rate of postoperative complications at two-week and six-month follow-up (Figure 4). At two-week follow-up, the highest complication rate was for patients with Hurley scale grade II, while the lowest was for patients with grade III. After six months, complications were observed only in patients with grade III (10.00%). All observed differences did not show statistical significance (*p* ≥ 0.050).

### 3.4. Type of Surgical Intervention

Among the factors analyzed, only the duration of hospitalization differentiated the groups of patients with common surgical interventions (Table 4). Patients treated with STSG had the longest hospitalization time (median ± QD: 10.00 ± 5.00), followed by co-graft ADM patients (7.00 ± 1.00). The median hospitalization time of rotational flap patients and rotational flap patients with additional PRP injection was identical (5.00 ± 1.00).

**Figure 5:**
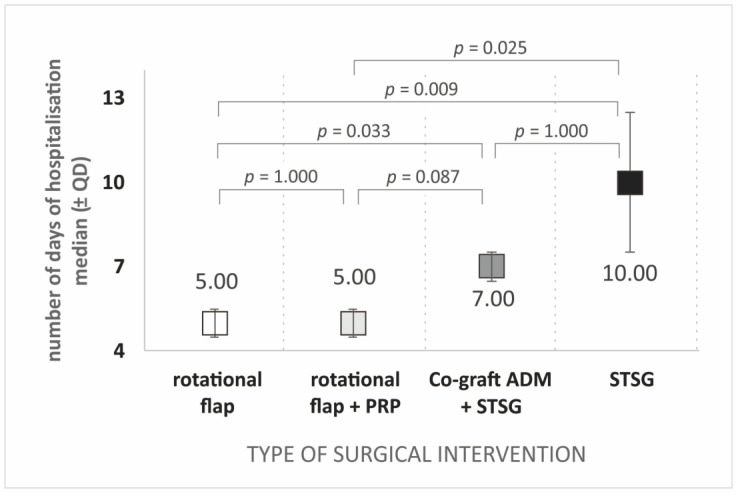
Duration of hospitalization depends on the type of surgical intervention. Legend: PRP, platelet-rich plasma; ADM, acellular dermal matrix; STSG, split-thickness skin graft.

Statistical differences in the duration of hospitalization according to the type of surgical intervention are shown in Figure 5.

**Figure 6:**
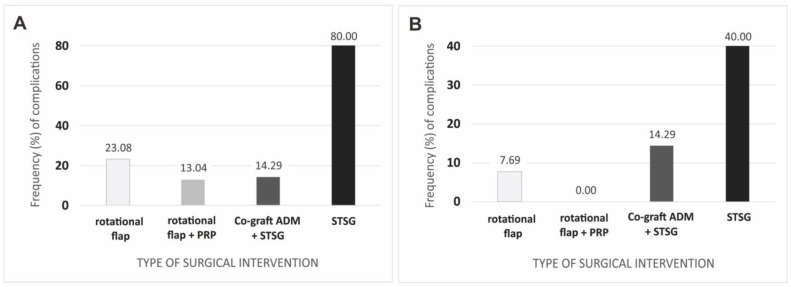
Complications after surgery regarding the type of surgical intervention: (**A**) in two weeks; and (**B**) a six-month follow-up. Legend: PRP, platelet-rich plasma; ADM, acellular dermal matrix; STSG, split-thickness skin graft.

It was also examined whether the type of surgical intervention affected the rate of postoperative complications. At two-week follow-up, the highest complication rate was in patients treated with STSG, while the lowest was in patients treated with rotational flap + PRP (Figure 6). After six months, complications were not observed only in the group of patients treated with rotational flap + PRP. As in the second week of follow-up, the highest complication rate was observed in the STSG-operated group. The rate of complications observed after surgery differentiated the groups of patients treated with each surgical method, both at two-week (*p* = 0.028) and six-month (*p* = 0.046) follow-up.

### 3.5. Cigarette Smoking

The time from first symptoms to final diagnosis and the duration of the disease did not differentiate patients who smoked from those who did not smoke (*p* = 0.744 and *p* = 0.367, respectively). The median time of hospitalization (±QD) was significantly longer in smokers relative to non-smokers (7.00 ± 2.00 vs. 5.00 ± 2.00, *p* = 0.009). As for blood parameters, nicotine smokers had higher median PLT levels (10^9^/l) than non-smokers (396.00 ± 140.50 vs. 317.00 ± 29.05), but these differences were not statistically significant (*p* = 0.051). As for the other parameters, the frequency of nicotinism did not differentiate patients with complications at the second week of follow-up from those without complications (25.53% vs. 23.08%, *p* = 0.856). Although the prevalence of nicotinism in patients with complications at the sixth postoperative month was higher than in those without complications (75.00% vs. 21.43%), these differences were not statistically significant and likely related to the low overall number of complications at six-month follow-up (n = 4). There were also no statistically significant differences in the incidence of nicotinism between patients with different Hurley’s stages of Hidradenitis suppurativa (*p* = 0.329) and the different surgical methods used (Table 5).

Patients with a vascular anomaly, as detected by Laser Speckle Contrast Analysis, had a lower prevalence of nicotinism than those without anomalies (11.11% vs. 25.81%), with the differences showing no statistical significance (*p* = 0.219).

## 4. Discussion

In our present study, we evaluated the effectiveness of standard reconstructive methods (local skin flaps, STSG) in wide excisions of HS lesions. We also compared modified methods of local skin flaps with PRP injections and co-grafts of ADM and STSG. We showed significant differences in terms of postoperative complications between the different groups of surgical intervention types. During the follow-up at two weeks after surgery, the highest number of postoperative complications was found in the group that operated only with the STSG method (80% complications). The most relevant comparison group for STSG is the ADM and STSG co-graft group, which showed a significantly lower complication rate (14.29%). ADM and STSG implanted concomitantly can improve healing significantly. Comparing the groups with local skin flaps, the present study showed a lower complication rate (13.04%) in the group with PRP injections compared to the group without PRP (23.08%). At 6-month follow-up after surgery, there were no postoperative complications in the PRP-injected group, and the differences in ADM and STSG co-grafts were also significantly lower than in the STSG alone group (14.29% vs. 40%). The results of our present study are very promising; however, the limitation is a small group. Further research on this matter should be conducted.

There are many studies concerning the healing process and the efficacy of autologous PRP [20,21]. De Angelis et al. conducted an in vitro and in vivo evaluation of a bio-functionalized scaffold composed of platelet-rich plasma (PRP) and hyaluronic acid (HA) used in 182 patients affected by chronic ulcers (diabetic and vascular), comparing the results with a control group of 182 patients treated with traditional dressings (HA alone). After 30 days, the patients who had undergone the combined treatment (PRP + HA) showed 96.8 ± 1.5% (± SD) re-epithelialization, as compared to 78.4 ± 4.4% (± SD) in the control group (HA only). Within 80 days, they had 98.4 ± 1.3% (± SD) re-epithelialization as compared to 87.8 ± 4.1% in the control group (HA only; *p* < 0.05) [21].

Much of the scientific work on PRP is about its regenerative effect on the skin; it is often used in dermatology and aesthetic medicine (skin rejuvenation, hair restoration) [21,22,23]. However, there is still a lack of literature in the field of HS surgery. Nicoli et al. presented a patient report with resection of severe HS of the nuchae and closure with PRP gel prepared with the RegenKit(^®^) to promote neovascularization and HPA, a delivery system for hyaluronic acid, to induce a neodermis at the wound bed and to stimulate regeneration in a humid and protected environment. Complete wound healing was achieved after 2 months [24]. The obtained result proved the efficacy of this treatment without complications. No recurrence was observed during the year after the surgical procedure. Severe HS can be safely and effectively managed with wide excision, PRP gel, and Hyalomatrix to achieve a successful outcome [24]. It’s difficult to compare the results of our study to those of Nicoli et al. due to methodological reasons; however, our results confirmed a good therapeutic effect in the PRP group (less surgical complication). However, the study of Nicoli et al. provided an interesting presentation of the usefulness of PRP in HS surgical treatment.

Our decision to use PRP injected into local skin flaps was dictated by a legitimate concern regarding the ischemia of skin flaps in this type of reconstruction. The literature reports many complications in skin flap reconstructions, but one of the most serious is flap ischemia [25,26,27].

In a mouse model, Rah et al. showed that PRP treatment enhanced the survival area and perfusion of the flap and reduced neutrophil accumulation in mice subjected to injury [28]. PRP treatment also showed a protective effect, with decreases in nitric oxide, myeloperoxidase, and malondialdehyde concentrations. Additionally, PRP suppressed monocyte chemotactic protein-1, TNF-α, IL-1β, and IL-6. Finally, PRP decreased ASK-1 and NF-κB expression in tissues with I/R injury. Rah et al. concluded that PRP acts as a protective factor during flap I/R injury by reducing reactive oxygen species levels and proinflammatory cytokines via decreased expression of pASK-1 and pNF-κB [26]. Wang et al. showed that platelet-rich plasma significantly improved flap survival rates of the PRP side flaps relative to the control in the 3, 7, and 14-day groups [29]. Histological analysis revealed significantly fewer inflammatory cells and an increased blood vessel density in the platelet-rich plasma side flap vs. the blank control side flap [30]. In the study by Wang et al., platelet-rich plasma significantly improved flap survival rates of the PRP side flaps relative to the control in the 3-day (74.4 ± 4.7% vs. 65.8 ± 6.8%; *p* < 0.05), 7-day (72.4 ± 7.5% vs. 58.5 ± 7.0%; *p* < 0.05), and 14-day (74.5 ± 5.0% vs. 65.0 ± 5.4%; *p* < 0.05) groups. Wang et al. concluded that platelet-rich plasma (PRP) promotes the survival of random rabbit flaps and, therefore, represents a promising treatment to prevent skin flap necrosis in reconstructive and plastic surgery [30]. In our study, in LASCA examinations, we found 10 patients with vascular anomalies in a group of PRP injections, but in only one patient did massive necrosis of the flap occur. The results of the presented study may show that our study confirms the previous results of Wang et al. on the rabbit model. These findings should be further explored in future studies. An interesting study by Takikawa et al. [31] was performed on rabbits in an in vivo model. Takikawa et al. injected PRP into the skin flaps 2 days before the surgery [31]. The authors conclude that, when PRP was administered 2 days before the flap elevation, improved flap survival was observed. The flap survival rate was significantly higher than those in the PRP (64.9 ± 4.0%) and control (61.2 ± 4.2%) groups. The pre-injection of PRP may thus represent a promising treatment to prevent skin flap necrosis in reconstructive surgery [31]. Our study showed that the hospitalization time in the rotational flap and PRP injection group was shorter than in the other groups, comparing the number of complications—the rotational flap and PRP injection group gave the fewest postoperative complications in the follow-up (13.04% complications in the group without PRP and 0% postoperative complications in the group with PRP injections).

In medical databases, there is still a lack of studies concerning ADM. The more valuable is the study by Lee YJ et al., which presents great results for ADM and STSG’s synergistic therapeutic effect in the treatment of deep tissue defects in the donor site of free flaps [32]. As in our study, Lee YJ et al. compared ADM + STSG to STSG alone. Their results showed that the Vancouver Scar Scale, vascularity sub-score (*p* = 0.003), and total score (*p* = 0.016) were significantly lower in the skin graft with the ADM group. Looking at the Patient and Observer Scar Assessment Scale, the pain (*p* = 0.037), stiffness sub-score (*p* = 0.002), and total score (*p* = 0.017) were found to be significantly lower in the skin graft with the ADM group [32]. The authors concluded that STSG with ADM results in better scar quality and fewer postoperative complications in objective and subjective terms, which is also a reflection of our study results. Chaffin et al. presented six patients that used ADM in HS treatment—probably the first use of ADM in the surgical treatment of HS in the literature. In four patients, ADM was followed by skin flap reconstruction. In two patients, ADM was used as a skin substitute. In one patient, 22 weeks after ADM application, STSG was used. However, the method of Chaffin et al. did not present an ADM and STSG co-graft [33]. The first presentation of co-grafted ADM and STSG in surgical treatment was performed by Gierek et al. [19]. In our previous manuscript, we evaluated good esthetic scar formation. The quality of life of each presented patient significantly improved [16]. As an additional observation in the present study, we can report that patients with the ADM and STSG co-graft have significantly better postoperative scars (very flexible scars) than the STSG group, which is similar to the results of our previous study describing this method [16]. Our study obviously has limitations in the number of groups—Co-graft ADM and STSG and STSG group—and it is difficult to draw firm conclusions from this comparison; however, we note a positive trend of better healing and shorter hospitalization time in the Co-graft ADM and STSG group (7 days vs. 10 days). Further studies with co-grafted ADM and STSG should be conducted in a separate study to evaluate this positive trend.

In summary, surgical treatment of HS is very challenging. It involves very extensive operations that require follow-up reconstructive methods. There are no ideal methods in such cases, but new solutions that improve existing methods must be sought. Such improvements to standard surgical methods are injections with PRP and co-grafted ADM and STSG.

## 5. Conclusions

The surgical treatment of large skin defects, as in the case of HS lesions, requires the use of modern reconstructive methods. Skin flaps and split-thickness skin grafts are proven methods, but with their limitations, standard reconstructive methods of closing large skin defects in the course of HS yield more postoperative complications than the new methods under investigation. The use of PRP injections in reconstructions (skin flaps) improved healing and reduced the number of complications, a notable trend in this study. Co-grafting ADM and STSG gave better therapeutic results than STSG alone (shorter hospitalization time, fewer postoperative complications). The results of this study should encourage surgeons to increase the use of PRP injection and ADM and STSG co-grafts in reconstructive surgery. However, further research should be conducted on this topic to improve surgical methods for the treatment of HS.

## Figures and Tables

**Table 1:** General and clinical characteristics of the study group.

Characteristic		
General	N (%)	61 (100.00)
	Age, median (±QD)	36.00 (7.50)
	Males, n (%)	29 (47.54)
	Females, n (%)	32 (52.46)
	BMI, median (±QD)	31.00 (3.80)
Blood parameters	PLT 10^9^/L, median (±QD)	322.50 (41.00)
	HGB g/dL, median (±QD)	12.80 (1.10)
	WBC 10^9^/L, median (±QD)	9.08 (2.09)
	MCH pg, median (±QD)	29.10 (2.03)
HS	Hurley’s stage of disease, n (%)	
	- I	5 (8.19)
	- II	6 (9.84)
	- III	50 (81.97)
	Location of HS, n (%)	
	- armpit	38 (62.29)
	- groin	15 (24.59)
	- buttocks	4 (6.56)
	- vulva	1 (1.64)
	- scrotum	1 (1.64)
	- submammary area	1 (1.64)
	- thigh	1 (1.64)
	Time from first symptoms to final diagnosis in years, median (±QD)	5.00 (5.00)
	Duration of disease in years, median (±QD)	7.00 (5.00)
Intervention	Type of surgical intervention, n (%)	
	- rotational flap	26 (42.62)
	- rotational flap + PRP	23 (37.70)
	- Co-graft ADM + STSG	7 (11.48)
	- STSG	5 (8.20)
	Time of hospitalization in days, median (±QD)	5.00 (1.50)
2 weeks follow-up	Healing normal, without complications, n (%)	47 (77.05)
	Wound dehiscence, n (%)	8 (13.11)
	Partial rejection of the graft/STSG n (%)	5 (8.20)
	Flap’s necrosis, n (%)	1 (1.64)
6 months follow-up	Wound healed completely, n (%)	55 (90.16)
	Keloid, n (%)	3 (4.92)
	Wound during the healing process, n (%)	2 (3.28)
	Wound healed completely with HS recurrence, n (%)	1 (1.64)
Comorbidities	Diabetes mellitus, n (%)	9 (14.75)
	Metabolic syndrome, n (%)	9 (14.75)
	Overweight/obesity (BMI ≥ 25), n (%)	46 (75.41)
	Obesity (BMI ≥ 30), n (%)	35 (57.38)
	Cigarette smoking, n (%)	15 (24.59)
	Hypertension, n (%)	16 (51.61)
	Hypothyroidism, n (%)	9 (14.75)
	Down’s syndrome, n (%)	1 (1.64)
	Asperger’s syndrome, n (%)	5 (8.20)

Legend: ADM, acellular dermal matrix; HS, hidradenitis suppurativa; PRP, platelet-rich plasma; STSG, split-thickness skin graft; QD, quartile deviation; VAC, vacuum-assisted closure. BMI, body mass index; PLT, platelets; HGB, hemoglobin; WBC, white blood cells; MCH, mean corpuscular hemoglobin.

**Table 2:** Spearman’s rank correlation coefficients (r_s_) between the analyzed factors.

	Age	BMI	Duration of Disease	Time from First Symptoms to Final Diagnosis	Time of Hospitalization
Age	-	0.31 *	0.66 *	0.5 9*	0.13
BMI	0.31 *	-	0.38 *	0.36 *	−0.19
Duration of disease	0.66 *	0.38 *	-	0.93 *	0.14
Time from first symptoms to final diagnosis	0.59 *	0.36 *	0.93*	-	0.09
Time of hospitalization	0.13	−0.19	0.14	0.09	-

* *p* < 0.050. Legend: BMI, body mass index.

**Table 3:** Clinical characteristics of patients with HS with regard to Hurley’s stage of Hidradenitis suppurativa.

Characteristic	Median ± QD			*p* Value			
	Hurley’s Stage of HS			Kruskal-Wallis	I vs. II	I vs. III	II vs. III
	I	II	III				
Age, years	22.00 ± 0.50	34.50 ± 9.50	37.50 ± 7.00	0.005	0.110	0.004	1.000
BMI, kg/(m)^2^	26.60 ± 4.35	33.40 ± 2.80	30.90 ± 4.30	0.313	-	-	-
PLT 10^9^/lL	247.00 ± 27.50	314.00 ± 59.50	324.00 ± 43.50	0.028	0.748	0.035	0.783
HGB g/dL	13.60 ± 1.80	14.10 ± 1.60	12.60 ± 1.10	0.144	-	-	-
WBC 10^9^/L	6.92 ± 0.57	8.81 ± 1.34	9.53 ± 2.36	0.127	-	-	-
MCH pg	29.60 ± 1.25	27.60 ± 1.70	29.10 ± 2.00	0.879	-	-	-
Time from first symptoms to final diagnosis, years	1.00 ± 0.50	8.50 ± 7.00	6.25 ± 4.00	0.026	0.120	0.023	1.000
Duration of disease, years	4.00 ± 1.50	9.50 ± 6.00	7.50 ± 4.50	0.047	0.272	0.041	1.000
Time of hospitalization, days	4.00 ± 1.00	5.00 ± 1.00	6.00 ± 1.50	0.062	-	-	-

Legend: BMI, body mass index; QD, quartile deviation; HS, hidradenitis suppurativa; PLT, platelets; HGB, hemoglobin; WBC, white blood cells; MCH, mean corpuscular hemoglobin.

**Table 4:** Clinical characteristics of patients regarding the type of surgical intervention.

Characteristic	Median ± QD				*p* Value
	Type of Surgical Intervention				Kruskal-Wallis
	Rotational Flap	Rotational Flap + PRP	Co-Graft ADM + STSG	STSG	
Age, years	38.00 ± 9.00	34.00 ± 10.00	35.00 ± 7.00	34.00 ± 3.50	0.927
BMI, kg/(m)^2^	31.38 ± 2.71	31.20 ± 4.80	24.00 ± 5.00	33.30 ± 4.20	0.448
PLT 10^9^/L	305.00 ± 32.00	338.00 ± 38.50	379.00 ± 132.50	299.50 ± 88.75	0.040 *
HGB g/dL	13.05 ± 1.05	12.50 ± 1.30	12.40 ± 1.50	13.00 ± 2.75	0.392
WBC 10^9^/L	7.70 ± 2.06	10.07 ± 1.57	10.35 ± 2.60	7.05 ± 0.56	0.010 *
MCH pg	29.50 ± 1.00	27.70 ± 2.50	26.40 ± 3.10	26.65 ± 1.30	0.118
Time from first symptoms to final diagnosis, years	7.00 ± 5.00	5.00 ± 5.25	5.00 ± 5.50	6.00 ± 4.00	0.781
Duration of disease, years	9.00 ± 4.50	7.00 ± 5.50	7.00 ± 5.00	8.00 ± 4.00	0.637
Time of hospitalization, days	5.00 ± 1.00	5.00 ± 1.00	7.00 ± 1.00	10.00 ± 5.00	0.001

Legend: PRP, platelet-rich plasma; ADM, acellular dermal matrix; STSG, split-thickness skin graft; BMI, body mass index; PLT, platelet; HGB, hemoglobin; WBC, white blood cells; MCH, mean corpuscular hemoglobin. *—not statistically significant (*p* ≥ 0.050) in post-hoc analysis.

**Table 5:** Distribution of cigarette smoking with regard to type of surgical intervention.

Characteristic	Type of Surgical Intervention				*p* Value
	Rotational Flap	Rotational Flap + PRP	Co-Graft ADM + STSG	STSG	
Cigarette smokers, % (n)	15.38 (4)	26.09 (6)	42.86 (3)	40.00 (2)	0.302
Non-smokers, % (n)	84.62 (22)	73.91 (23)	57.14 (4)	60.00 (3)	

Legend: PRP, platelet-rich plasma; ADM, acellular dermal matrix; STSG, split-thickness skin graft.

## Data Availability

Not applicable.

## Supplementary Materials

The following supporting information can be downloaded at: https://www.mdpi.com/article/10.3390/jcm12062112/s1. Table S1: STROBE statement.
